# Comprehensive transcriptomic and metabolomic analysis revealed drought tolerance regulatory pathways in upland cotton

**DOI:** 10.3389/fpls.2025.1571944

**Published:** 2025-04-17

**Authors:** Fenglei Sun, Junhao Wang, Shiwei Geng, Yajun Liang, Zhaolong Gong, Ni Yang, Shuaishuai Qian, Nala Zhang, Xueyuan Li, Junduo Wang, Juyun Zheng

**Affiliations:** ^1^ Cotton Research Institute of Xinjiang Uygur Autonomous Region Academy of Agricultural Sciences/Xinjiang Key Laboratory of Cotton Genetic Improvement and Intelligent Production, Urumqi, Xinjiang, China; ^2^ Engineering Research Centre of Cotton, Ministry of Education/College of Agriculture, Xinjiang Agricultural University, Urumqi, Xinjiang, China; ^3^ National Key Laboratory of Crop Genetic Improvement, Hubei Hong Shan Laboratory, Huazhong Agricultural University, Wuhan, China

**Keywords:** cotton, drought stress, transcriptomics, metabolomics, multi-omics analysis

## Abstract

Cotton is a kind of cash crop widely planted in arid and semi-arid areas. In this study, we performed multi-omics analysis of two drought resistant extreme materials, Yumian 4 and C460, under drought stress. Transcriptome analysis showed that DY (post-drought stress Yumian 4) had more differentially expressed genes than DC (post-drought stress C460), and there were 10247 DEGs in the two comparison groups. Metabolomics analysis identified 1766 metabolites, which were divided into 12 classes. The up-regulated metabolites mainly included lipid accumulation, phenylpropanoid biosynthesis, and flavonoids. The combined transcriptome and metabolome analysis highlighted the importance of phenylpropanoid biosynthesis in enhancing drought tolerance. Combining the two omics analysis, it was found that the enrichment pathway of differential genes and differential metabolites is mainly in the phenylpropane biosynthesis pathway, which contains 23 related candidate genes. In summary, the results of multi-omics analysis of the two extreme drought resistance cotton materials showed that they enhanced drought resistance by affecting phenylpropanoid biosynthesis pathways. Promote the accumulation of osmotic substances. The results further deepen our understanding of the molecular mechanism of drought tolerance in cotton and provide new insights for molecular breeding of cotton.

## Introduction

Cotton is one of the most important natural fiber crops in the world at present, which has an extremely important impact on domestic textile industry and agricultural economy. Our country is the world’s largest producer of cotton, according to the statistics of relevant departments, our country cotton planting area, per unit yield and total output are in the forefront of the world. Therefore, cotton production also has an important impact on the development of the national economy (Sun et al., 2021).

Drought stress is one of the major abiotic stresses faced by plants under global climate change, which has significant effects on physiological indexes of different plants. In recent years, studies have shown that drought stress can significantly reduce the photosynthetic rate of plants, affect water use efficiency, and lead to the accumulation of osmoregulatory substances such as proline and soluble sugar (Muhammad et al., 2018; Aditi et al., 2020). In wheat, drought stress also causes changes in antioxidant enzyme activity in response to oxidative stress (Zulfiqar et al., 2024). In addition, different plant species have different responses to drought stress, for example, maize and rice show different stomatal conductance regulation strategies under drought. Transcriptomic studies have revealed differentially expressed genes under drought stress, which are involved in signal transduction, transcriptional regulation and metabolic pathways (Fidel et al., 2022; Niu et al., 2023). miRNA are also involved in drought stress response and affect plant physiological processes by regulating the expression of target genes (Waqar et al., 2022; Eom et al., 2019).

In recent years, omics technology has become an important means to analyze the drought stress response mechanism of crops (Li et al., 2021). Transcriptomic studies have shown that a large number of genes are differentially expressed in crops under drought stress, involving signal transduction, transcriptional regulation and metabolic pathways (Zhu et al., 2019; Jiang et al., 2023). Proteomic analysis further revealed the key proteins of drought response, such as osmoregulatory proteins and antioxidant enzymes (Gao et al., 2023; Ren et al., 2022). Metabolomics studies have revealed the changes of metabolites in crops under drought stress, providing a new perspective for understanding the biochemical basis of drought response (Michael et al., 2020; Wang et al., 2023). In addition, epigenetic studies have shown that drought can cause epigenetic changes such as DNA methylation and histone modification, affecting gene expression (Chang et al., 2023; Wang et al., 2021). The comprehensive application of these omics techniques provided strong support for the in-depth analysis of drought stress response mechanism of crops and the cultivation of drought-tolerant varieties. Transcriptome analysis of rice under drought stress using RNA-Seq technology revealed a number of differentially expressed genes related to drought response, including genes involved in osmoregulation, antioxidant defense and signal transduction pathways (Anuj et al., 2023). The expression changes of these genes provide important clues for understanding the molecular mechanism of drought tolerance in rice. Protein expression profiles of wheat leaves under drought stress were analyzed by ITRAQ-labeled proteomics technology, and several proteins related to drought tolerance were identified, such as osmoregulatory proteins, molecular chaperones and heat shock proteins (Wang et al., 2019). These proteins perform important functions under drought stress, helping to maintain cell homeostasis and protect cell structure. Using GC-MS and LC-MS techniques to study the changes of metabolites in maize under drought stress, the researchers identified a series of metabolites related to drought response, including amino acids, organic acids and sugars (Zhang et al., 2021). The accumulation or consumption of these metabolites reflected the adjustment of metabolic pathway of maize under drought stress, and provided the basis of metabolic level for breeding drought-tolerant varieties. Drought stress can cause changes in DNA methylation patterns and affect gene expression. The researchers used sequencing techniques to analyze changes in DNA methylation levels in wheat under drought stress and found a number of differentially methylated regions associated with drought response (Salehe et al., 2024). These results indicate that multi-omics analysis plays an important role in drought tolerance of crops.

In recent years, the phenylpropane pathway has played an important role in plant response to drought stress. Studies have shown that it plays a crucial role in enhancing plant tolerance by producing secondary metabolites. Drought stress can significantly induce the expression of phenylpropane-related genes and enzyme activity, thus promoting the synthesis of secondary metabolites and improving drought resistance of plants (Ge et al., 2023; Wang et al., 2024). The expressions of phenylalanine aminolyase (PAL), C4H and 4-coumarine-CoA ligase (4CL) were significantly up-regulated under drought conditions (Ge et al., 2023; Wang et al., 2024). These enzymes catalyze the synthesis of lignin and flavonoids, which contribute to cell wall reinforcement and antioxidant defense mechanisms, respectively. Lignin pathway and flavonoid pathway are two important branches of phenylpropane metabolism. Lignin, the second most abundant polymer on Earth, accumulates mainly in the secondary cell walls of plants, provides mechanical support for plants, and is involved in the formation of conduits, critical for the transport of water and mineral elements (Ge et al., 2023; Vanholme et al., 2010). Flavonoids are the most diverse metabolites in phenylpropane metabolic pathway, including flavonoids, flavonols, flavanones, isoflavones, anthocyanins, proanthocyanidins and other compounds, which play an important role in plant flower color formation, pollination insect attraction, antioxidant defense and other aspects (Ge et al., 2023; Winkel-Shirley, 2001). In addition, the phenylpropane pathway is involved in drought signal transduction and response by regulating the expression of related transcription factors and genes. For example, MYB transcription factor family can regulate the expression of phenylpropane pathway related genes under drought stress, thus affecting drought resistance of plants (Guan et al., 2020).

In this study, transcriptome and metabolomics were used for multi-omics analysis to explore the regulatory mechanism of cotton response to drought stress. The results of this study are of great significance for exploring the response of cotton to water stress, helping to clarify the regulatory network of plant response to drought stress, and providing valuable information for breeding new varieties of drought-tolerant cotton.

## Materials and methods

### Material planting and drought treatment

Drought-tolerant variety C460 (R) and drought-sensitive variety Yumian 4 (S) were identified by the previous research group through drought stress screening. In this study, R and S were used as experimental materials to carry out hydroponics experiments. First of all, the two materials were cultured in the germination box until the seeds were white, and the seeds with uniform growth were selected and uniformly transplanted to the hydroponic pot. After cultivation to the trifoliate stage, PEG (15%) stress treatments were applied, and true leaves of R and S cotton were collected at 4, 8, 12, and 24 h after stress treatment, and three duplicate samples were collected for each treatment time. After rapid freezing in liquid nitrogen, it is temporarily stored in a refrigerator at -80°C for subsequent use.

### Transcriptome sequencing analysis

Total RNA was extracted by TRIzol method for subsequent analysis, and RNA integrity was measured by Agilent 2100 bioanalyzer (Agilent Technologies, Santa Clara, CA, USA). Total RNA was enriched by mRNA using Oligo dT magnetic beads. After fragmentation, the cDNA is synthesized, its end is modified, and the cDNA library is purified. Sequencing by Illumina platform. Raw readings are processed using software to obtain clean readings (Chen et al., 2018). Software quickly and accurately compares clean readings to reference genomes and predicts new genes (Kim et al., 2015; Pertea et al., 2015). According to the result of comparison with the reference genome, the read number of genes was obtained through the software, and then the expression level of gene FPKM was obtained (Wang et al., 2014; Liao et al., 2014). DEGs with FDR ≤ 0.05 and |log2FoldChange|≥ 1 were identified by DESeq2 (1.20.0) software as the criteria for differential gene screening. GO functional enrichment analysis and KEGG pathway enrichment analysis were performed on DEGs by software, and genes with p values of <0.05 were considered to be significantly enriched (Young et al., 2010; Ogata et al., 1999). The accuracy of transcriptomic analyses was analyzed using Pearson correlation coefficient and principal component analysis (**Supplementary Figures S1**) (Robinson et al., 2010).

### Metabolomics sequencing analysis

This study is based on liquid chromatography-mass spectrometry (LC-MS) technology for non-targeted metabolomics studies (Want et al., 2010). Simple screening of metabolites by Compound Discoverer 3.3 (CD3.3, Thermo Fisher, Waltham, MA, USA) using raw data. The metabolites were then compared with mzCloud (https://www.mzcloud.org/), mzVault, and MassList databases for secondary identification. Finally, metabolites with coefficient of variance (CV) less than 30% were used for follow-up analysis (Dai et al., 2017). The identified metabolites were annotated by KEGG database for functional characterization. DAM screening is mainly based on variable importance (VIP), multiple change (FC) and P-value (Haspel et al., 2014). The threshold is set to VIP > 1.0, p < 0.05, FC > 1.2, or FC < 0.833.

### Statistical analysis

One-way ANOVA was used to analyze the significance of phenotypic difference of drought-treated cotton (0, 4, 8, 12 and 24 h). Student t test was used to detect significant differences in gene expression under drought treatment (0, 4, 8, 12 and 24 h). IBM SPSS Statistics v21 software was used to conduct one-way ANOVA and Student t test.

## Result

Analysis of differentially expressed genes (DEGs) in cotton under drought stress

**Figure 1:**
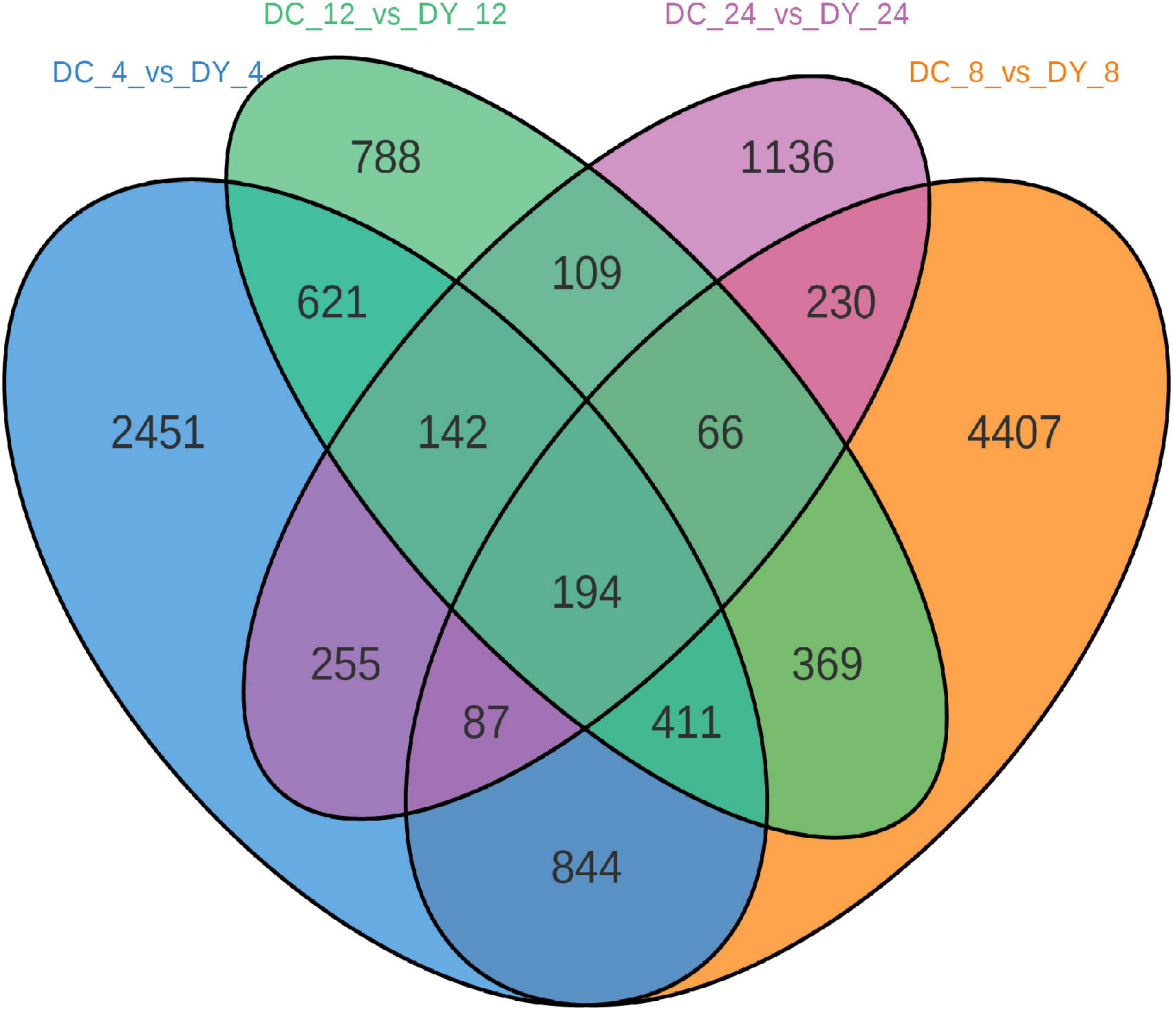
Venn diagram of differential genes.

All the samples were divided into ten groups: they included Yumian 4 and C460 control groups (CK_Y and CK_C), and drought stress treatment groups for 4h, 8h, 12h and 24h (DY_4, DY_8, DY_12, DY_24, DC_4, DC_8, DC_12 and DC_24). A total of 210.83 GB of clean sequencing data were obtained (**Supplementary Table S1**). The genome alignment rate was greater than 90%, the Pearson correlation coefficient (R^2^) of the biorepeats was greater than 0.9 in the inter-group comparisons (**Supplementary Figure S1A**), and there were significant differences in gene expression between groups (**Supplementary Figure S1B**). When we compared the DEGs of DC_4 and DY_4, DC_8 and DY_8, DC_12 and DY_12, and DC_24 and DY_24 (**Figure 1**), we found that 194 DEGs of the two varieties were expressed under drought stress. It is hypothesized that these genes are involved in the drought stress response of cotton and are closely related to its tolerance to drought. In DC_12 and DY_12 and DC_24 and DY_24, only a small part of DEGs were common in drought stress, and the number of genes induced by DC_4 and DY_4 and DC_8 and DY_8 were more.

### Functional analysis of DEGs under drought stress

**Figure 2:**
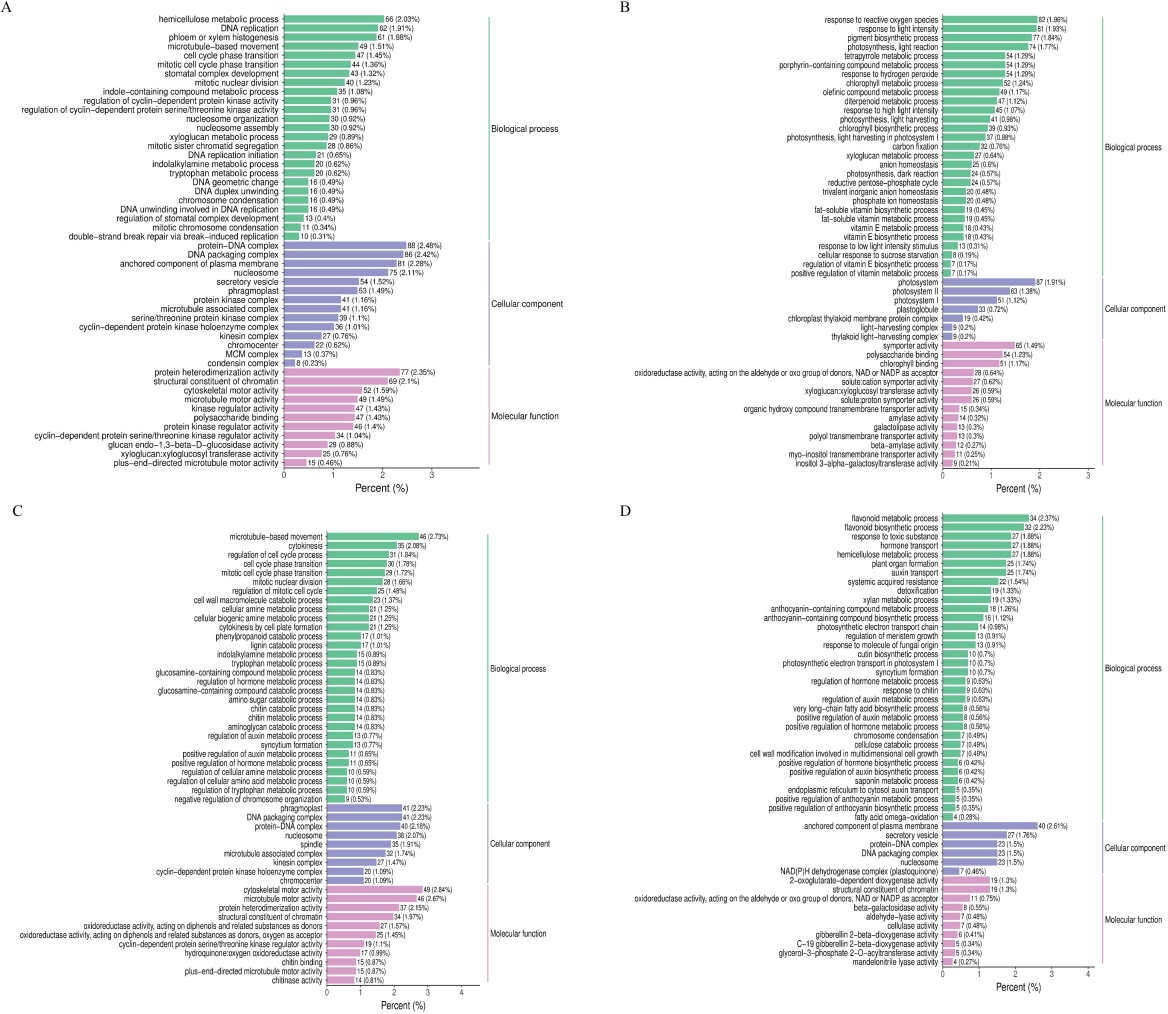
GO enrichment analysis of DEG **(A)** Yumian 4 and C460 treated with drought stress for 4h; **(B)** Yumian 4 and C460 treated with drought stress for 8h; **(C)** Yumian 4 and C460 treated with drought stress for 12h; **(D)** Yumian 4 and C460 treated with drought stress for 24h).

**Figure 3:**
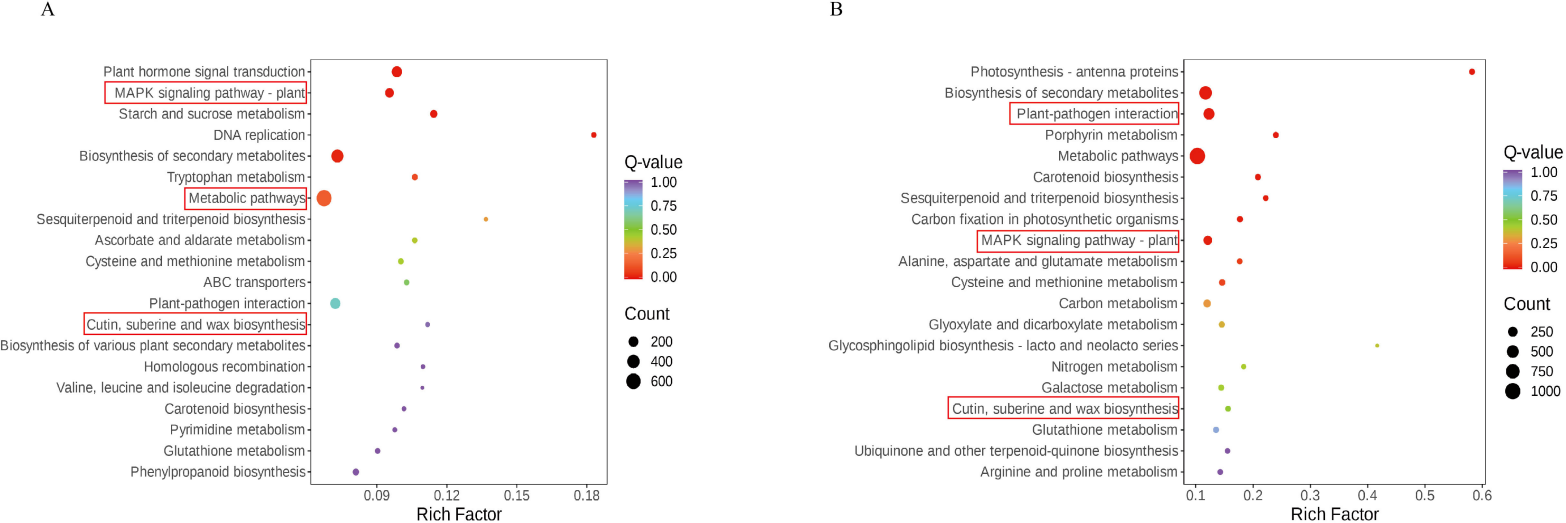
KEGG rich distribution point diagram **(A)** Yumian 4 and C460 treated with drought stress for 4h; **(B)** Yumian 4 and C460 treated with drought stress for 8h).

In order to further explore the function of DEGs under drought stress and analyze the response mechanism of cotton to drought stress, GO and KEGG analyses were performed on the up-regulated DEGs in R and the down-regulated DEGs in S (**Figure 2**). DEGs in the four comparison groups (DC_4 and DY_4, DC_8 and DY_8, DC_12 and DY_12, and DC_24 and DY_24) mainly concentrated in three categories: biological processes (BPs), molecular functions (MFs), and cell components (CCs) (**Figure 2**). Up-regulated DEGs is mainly involved in the negative regulation of biosynthesis and hormone metabolism (BPs), photosynthesis and microtubule associated complexes (MFs), redox enzyme activity and xylosyltransferase activity (CCs). Down-regulated DEGs are mainly involved in chromosome condensation (BPs), driver protein complexes (MFs), and chromatin structural components (CCs). KEGG enrichment analysis showed that the top 20 metabolic pathways enriched after drought treatment included the biosynthesis of secondary metabolites. Biosynthesis of cutin, wax and wax; Cysteine and methionine metabolism; Biosynthesis of glucosinolate; MAPK- signaling pathway (**Figure 3**). “MAPK- signaling pathway”, “keratin, wax and wax biosynthesis” and “plant hormone signal transduction” were significantly enriched in CK *vs* D. The biological activity of cotton seedlings under drought stress may be mainly maintained by regulating the biosynthesis of secondary metabolites, and further may be mainly dependent on enhancing the biosynthesis of cutin, wax and wax and plant hormone signal transduction.

In DC_4 and DY_4 and DC_8 and DY_8, DEGs were significantly enriched in 51 pathways (p < 0.05), including plant hormone signal transduction, MAPK signal pathway, starch and sucrose metabolism, keratin, wax and wax biosynthesis, glutathione metabolism, ascorbic acid and uronate metabolism. Among them, “plant hormone signal transduction”, “starch and sucrose metabolism” and “ascorbic acid and uronate metabolism” were only enriched in DC_4 and DY_4. The rest were enriched in DC_8 and DY_8. The top 20 most significantly enriched pathways in both comparisons are shown in **Figure 3**.

### Analysis of cotton metabolites under drought stress

**Figure 4:**
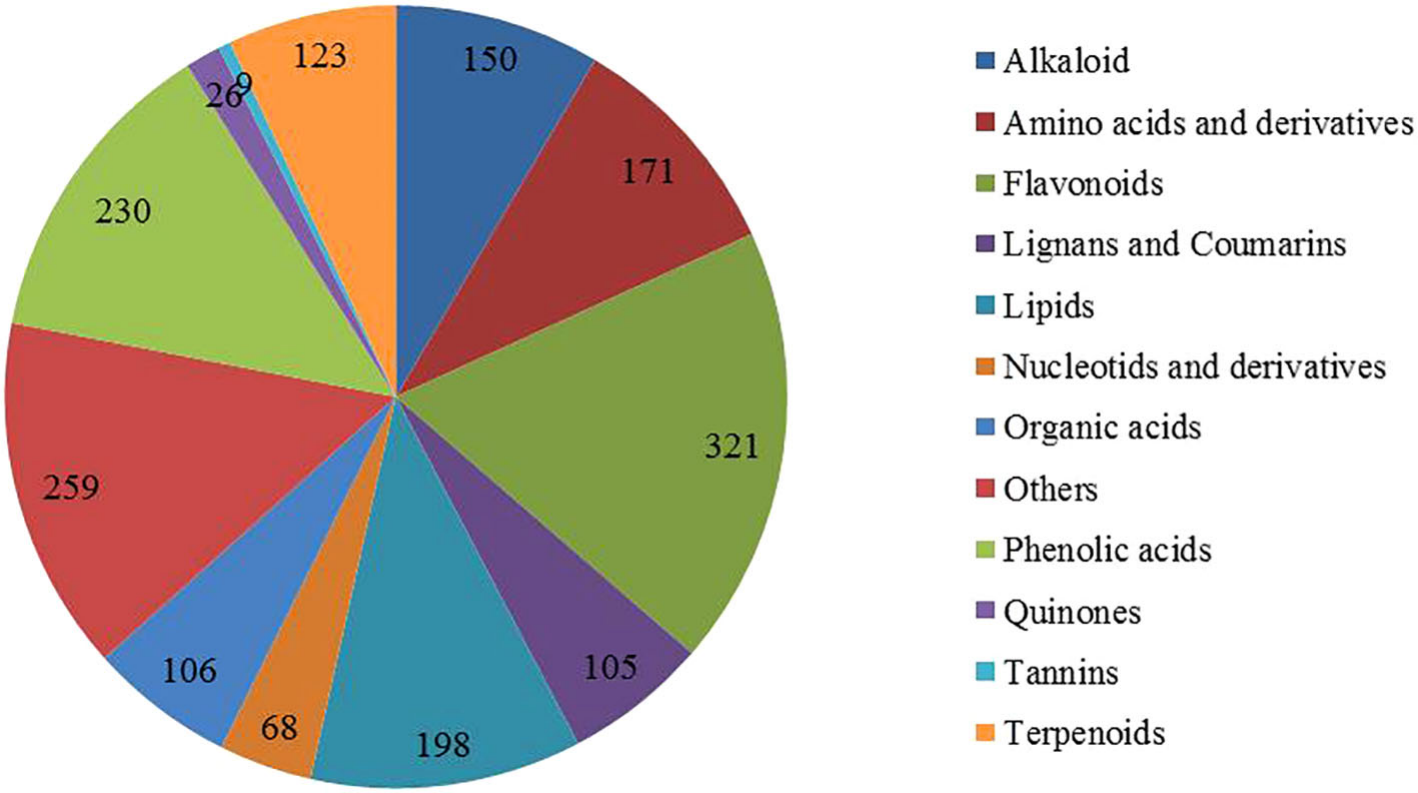
Pie chart of different metabolite content.

In order to explore the regulatory mechanism of cotton response to drought stress, metabolomics technology was used to analyze and identify the metabolites after drought stress. A total of 1766 metabolites were detected, which were divided into 12 categories, including 198 lipids, 321 flavonoids, 230 phenolic acids, 171 amino acids and their derivatives, 150 alkaloids, 68 nucleic acids and their derivatives, 106 organic acids, 105 lignins and coumarins, 123 terpenoids, 26 quinones, and 9 tannins, and 259 other substances (**Figure 4**). PCA showed significant interspecific differences between drought treatment (group D) and control (group CK) (**Supplementary Figure S2**), indicating that seedlings were slightly damaged at 4 h and significantly damaged at 8 h under drought stress, which was consistent with transcriptomic results.

Orthogonal partial least squares discriminant analysis (OPLS-DA) was used to mine differential metabolites, and the contribution of each metabolite in the OPLS-DA model was assessed by variable importance (VIP). |log2FC| ≥ 1 and VIP ≥ 1 were used as thresholds for screening. In DC_4 and DY_4, 137 important differentially expressed metabolites (DAMs) were screened, of which 57 were up-regulated and 80 were down-regulated. We found that four categories contained more than 10 DAMs: phenolic acids (20,14.6%), flavonoids (48,35.0%), alkaloids (13,9.5%), and terpenoids (12,8.8%). There are 291 important DAMs in DC_8 and DY_8, 233 of which are up-regulated and 68 down-regulated. Similarly, six classes contained more than 10 DAMs: lipids (36,12.4%), flavonoids (109, 37.5%), and phenolic acids (33,11.3%) etc. In DC_12 and DY_12, 222 DAMs were screened, of which 118 DAMS were up-regulated and 104 DAMS were down-regulated. There are eight categories that contain more than 10 DAMs: phenolic acids (35,15.8%), flavonoids (76,34.2%), and lipids (10,4.5%) etc. There are 143 important DAMs in DC_24 and DY_24, 43 of which are up-regulated and 100 down-regulated. There were four classes containing more than 10 DAMs: phenolic acids (25,17.5%), flavonoids (46,32.2%), and terpenes (10, 7.0%) etc.

**Figure 5:**
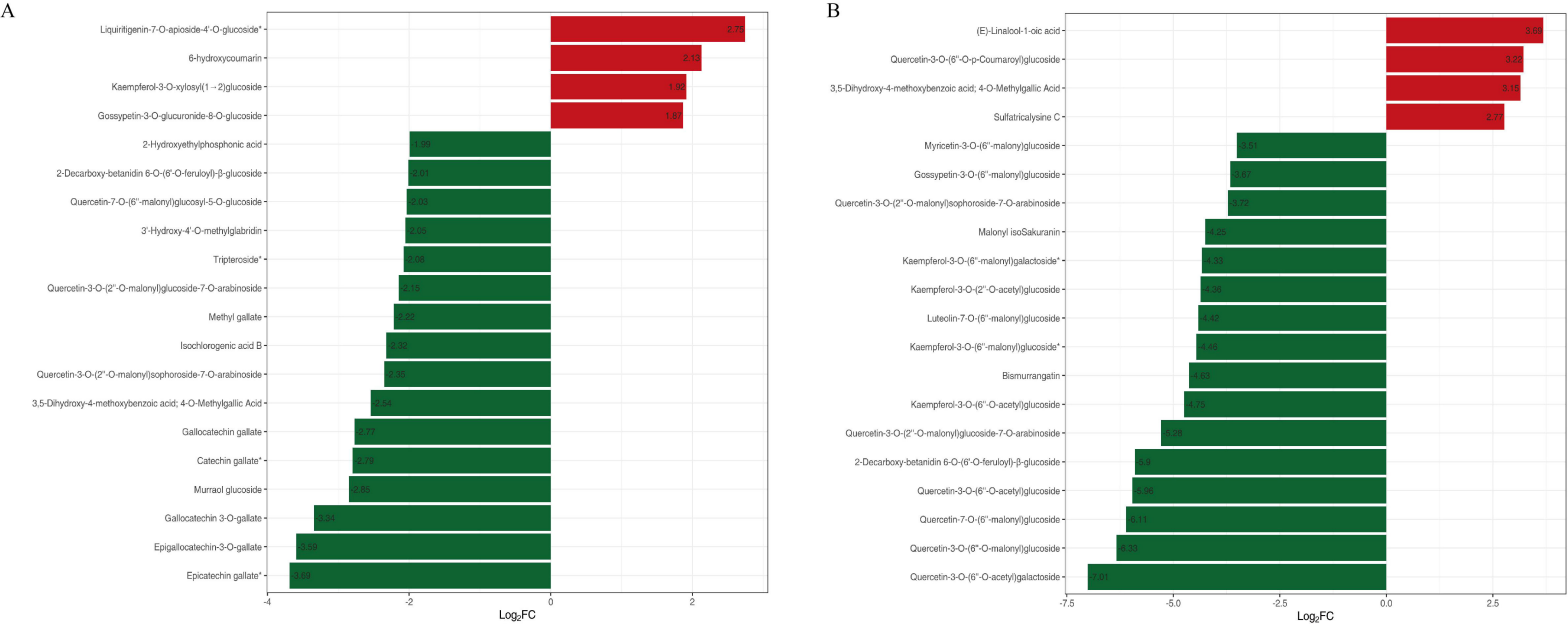
Differential multiplex histogram of DAMs **(A)** Yumian 4 and C460 treated with drought stress for 4h; **(B)** Yumian 4 and C460 treated with drought stress for 8h).

There were significant changes in the abundance of 428 metabolites in DC_4 and DY_4 and DC_8 and DY_8 groups. Compared with drought-tolerant varieties, the metabolite abundance of drought-sensitive varieties varied greatly, in which the increase of lipids was the largest, and the decrease of flavonoids was the largest. Based on the multiples of metabolite accumulation, we identified the top 10 DAMs that increased or decreased in control and drought treatment (**Figures 5 A, B**). Through screening, we found that the co-accumulation of 39 metabolites changed in all groups (lipids were the substances with the main accumulation increases, while flavonoids and phenolic acids were reduced). Notably, most of the different substances that accumulate in drought-tolerant varieties are related to energy metabolism (**Figures 5 A, B**).

**Figure 6:**
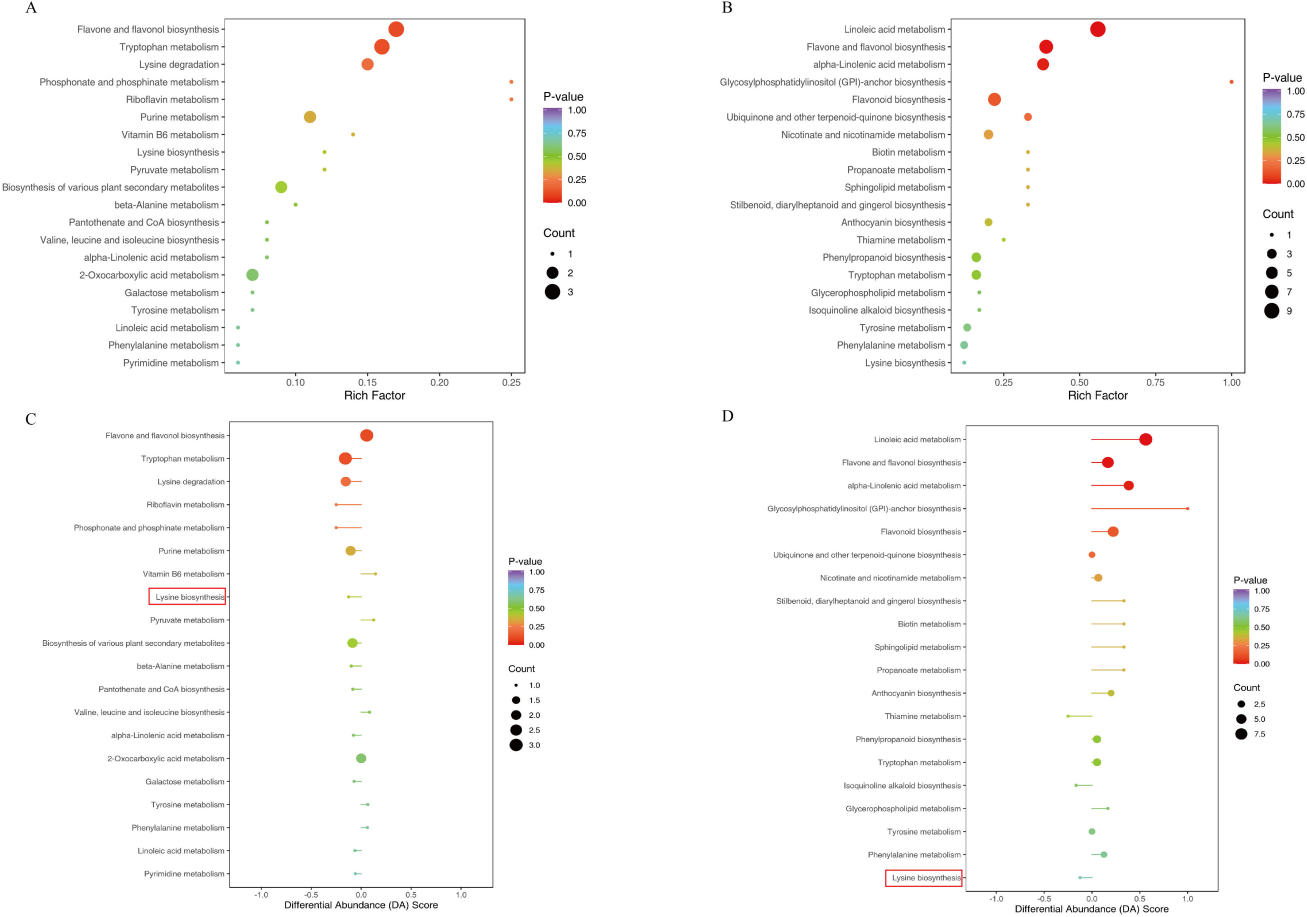
KEGG enrichment analysis of differential metabolites is shown in **(A, B) (A)** Yumian 4 and C460 treated with drought stress for 4h; **(B)** Yumian 4 and C460 treated with drought stress for 8h). **(C, D)** was the differential abundances of differential metabolites. **(C)** Yumian 4 and C460 treated with drought stress for 4h; **(D)** Yumian 4 and C460 treated with drought stress for 8h).

DAMs were enriched into KEGG pathway, and p<0.05 was used as the screening condition. The results showed that the carbon metabolism, arginine biosynthesis, linoleic acid metabolism, phenylpropane biosynthesis, plant hormone signal transduction and carotenoid biosynthesis of the two varieties were significantly enriched under drought. Metabolism of alanine, aspartate and glutamate, isoflavone biosynthesis, folate biosynthesis, flavonoids and flavonols biosynthesis, and tryptophan metabolism are only enriched in CK_C *vs* DC. Pentose phosphate pathway, D-amino acid metabolism, glyoxylate and dicarboxylate metabolism, purine metabolism, unsaturated fatty acid biosynthesis, and 2-oxycarboxylic acid metabolism are associated with more abundant DAMs in CK_Y *vs* DY (**Figures 6A, B**). The common enrichment pathways of DC_4 *vs* DY_4 and DC_8 *vs* DY_8 are as follows: Biosynthesis of flavonoids and flavonols; phenylalanine metabolism and Lysine biosynthesis, etc. In order to elucidate the overall trend of KEGG metabolic pathways, we performed differential abundance analysis of the DC_4 *vs* DY_4 and DC_8 *vs* DY_8 pathway maps (**Figures 6C, D**). The results showed that lysine biosynthesis was significantly down-regulated.

**Figure 7:**
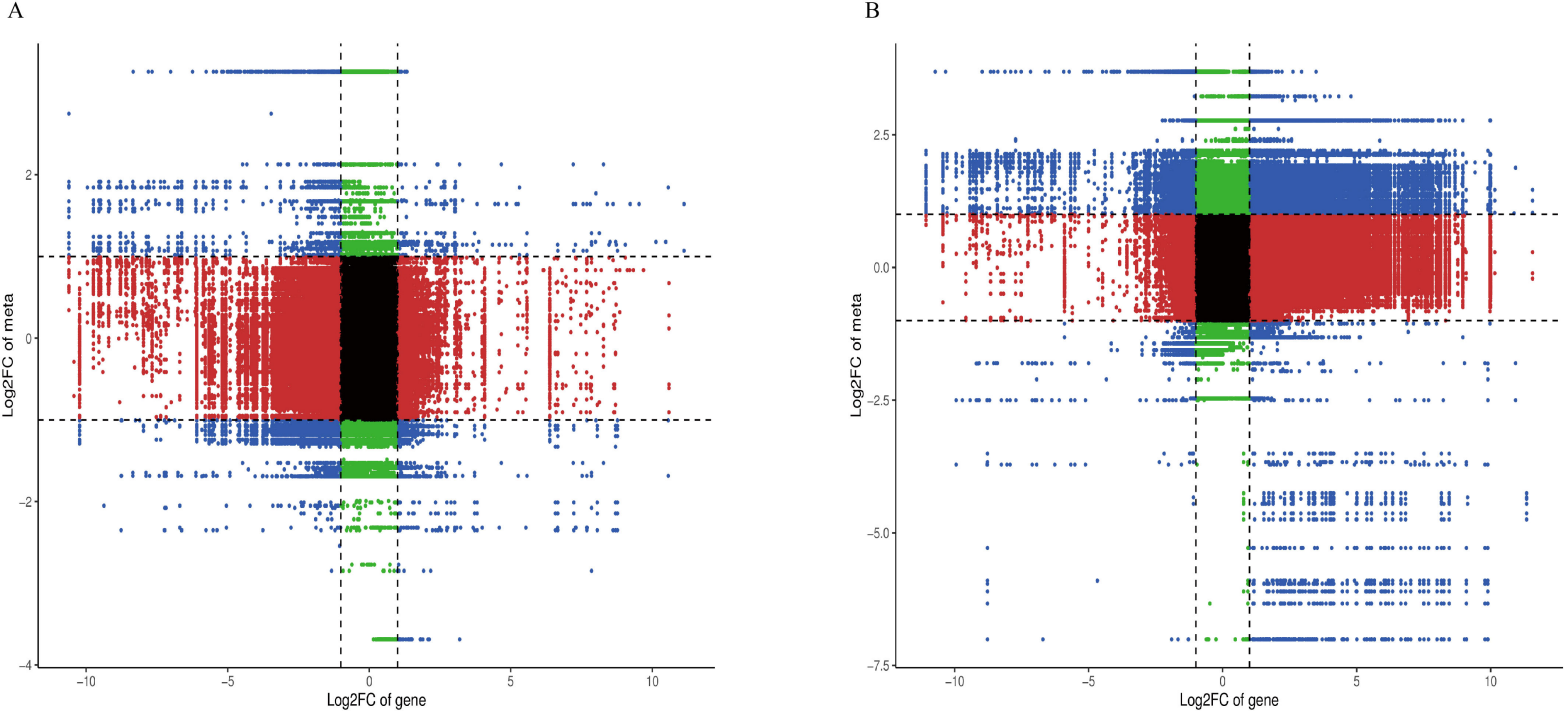
Combined gene and metabolite analysis **(A)** Yumian 4 and C460 treated with drought stress for 4h; **(B)** Yumian 4 and C460 treated with drought stress for 8h).

DEGs and DAMs were integrated into the KEGG pathway, and the enrichment pathway of both DEGs and DAMs was screened using p < 0.05 as the threshold value. The enrichment pathways in DC_4 *vs* DY_4 and DC_8 *vs* DY_8 are ABC transporters, biosynthesis of secondary metabolites, phenylpropane biosynthesis, amino acid biosynthesis, and biosynthesis of various plant secondary metabolites. A nine-quadrant map of DEG and DAM correlation based on correlation value > 0.8 shows that most DEG is consistent with DAM pattern, with genes up-regulated, metabolites remaining unchanged or down-regulated, and positive gene regulation predominates over negative regulation among genes affecting metabolic changes (**Figures 7A, B**).

**Figure 8:**
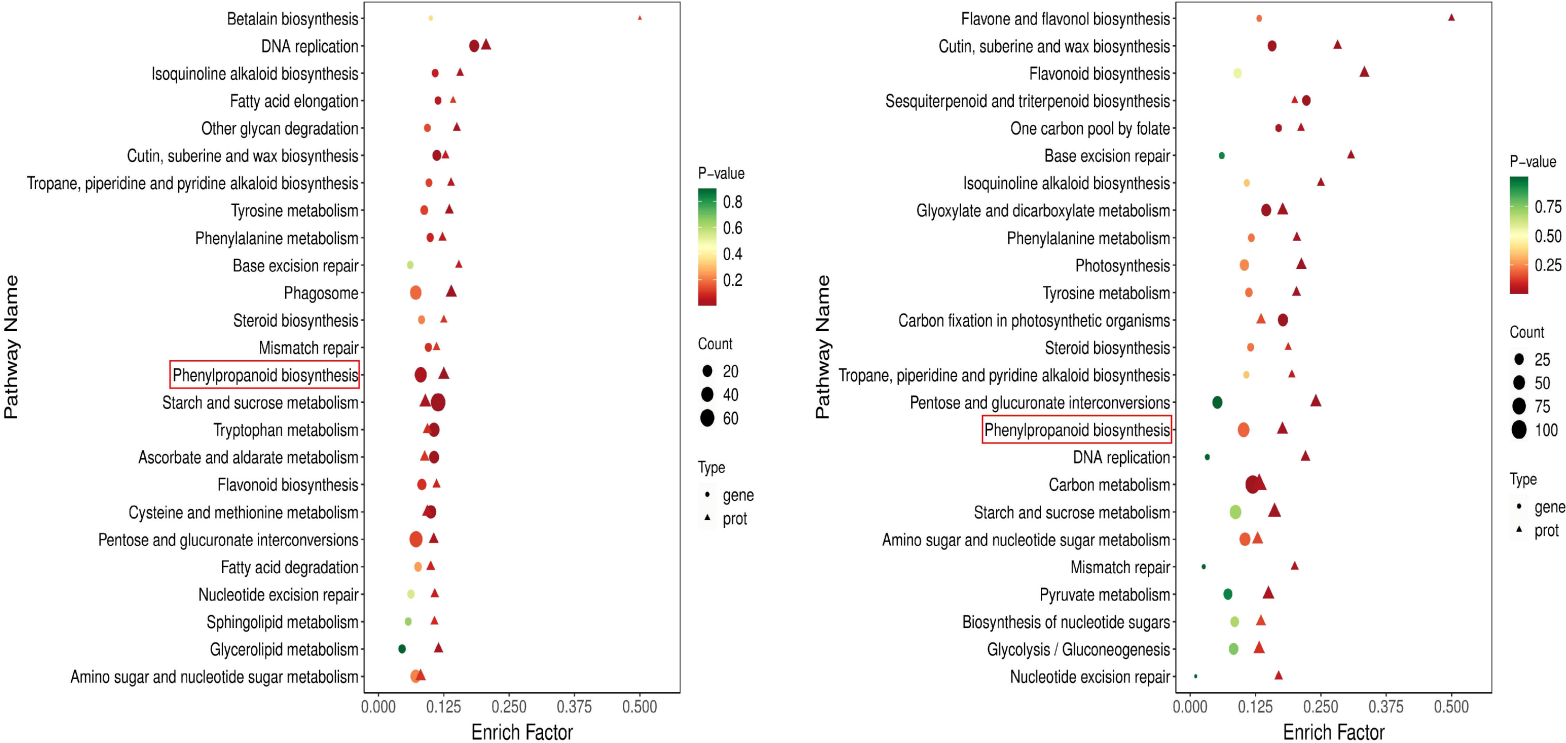
Multi-omics KEGG enrichment analysis **(A)** Yumian 4 and C460 treated with drought stress for 4h; **(B)** Yumian 4 and C460 treated with drought stress for 8h).

Under drought conditions, plants will synthesize a large number of cell transport enzymes and antioxidant enzymes to enhance their drought tolerance. Therefore, next, we use the comprehensive analysis data of transcriptome and metabolome to focus on the change trend of cotton transport enzymes and oxidase under drought conditions. In DC_4 and DY_4 and DC_8 and DY_8, ABC transporters and phenylpropane biosynthetic pathways were significantly enriched at both transcriptome and metabolome levels (p ≤ 0.01). Caffeic acid 3-O-methyltransferase, members of the ABC transporter G family, β-Amyrin11-oxidase, and 1-deoxyd-xylose-5-phosphate synthetase were significantly increased in both treatments (**Figures 8A, B**).

### Effects of drought stress on phenylpropane biosynthesis pathways in cotton

**Figure 9:**
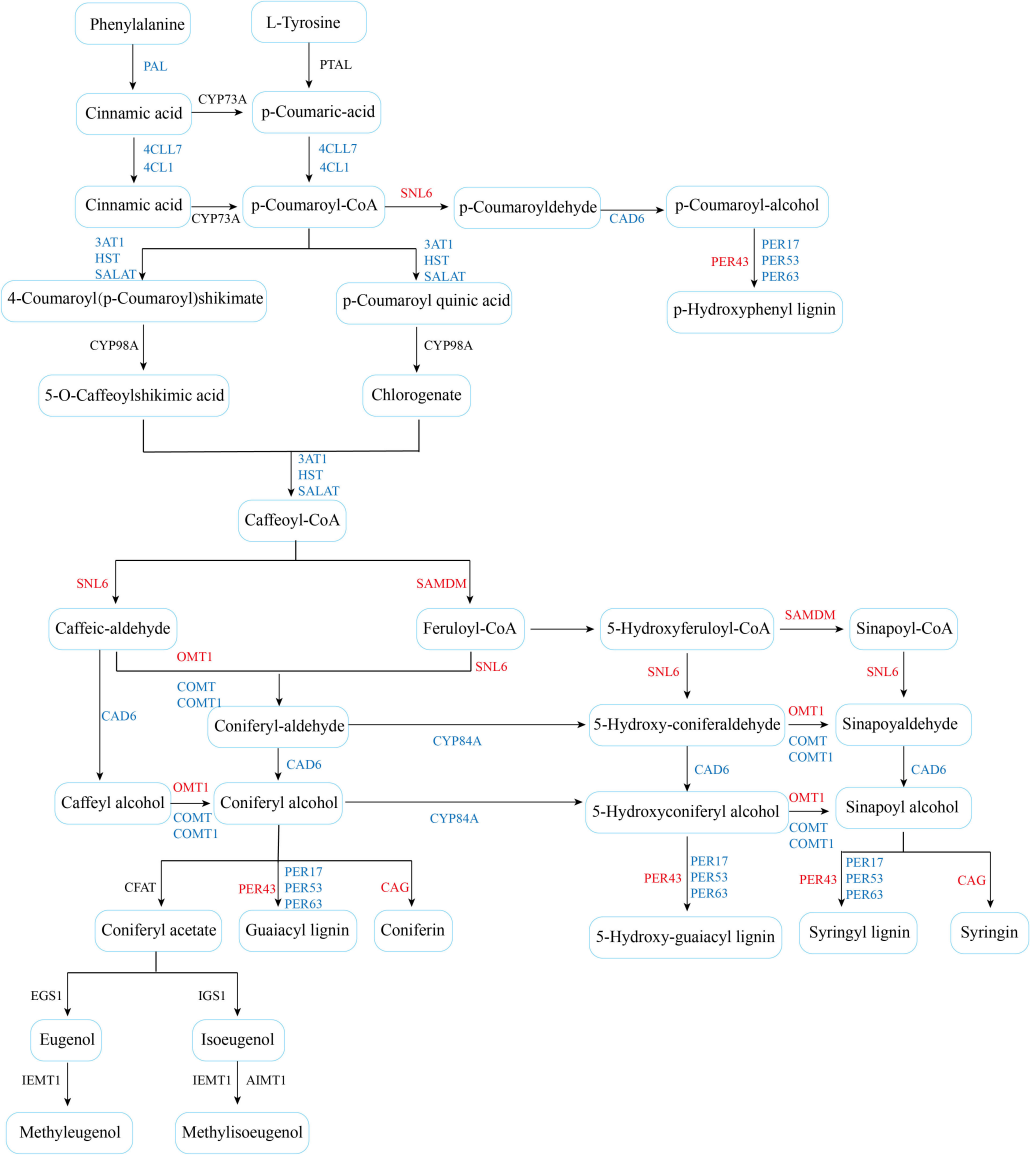
Pathways of phenylpropane biosynthesis in cotton under drought stress.

**Figure 10:**
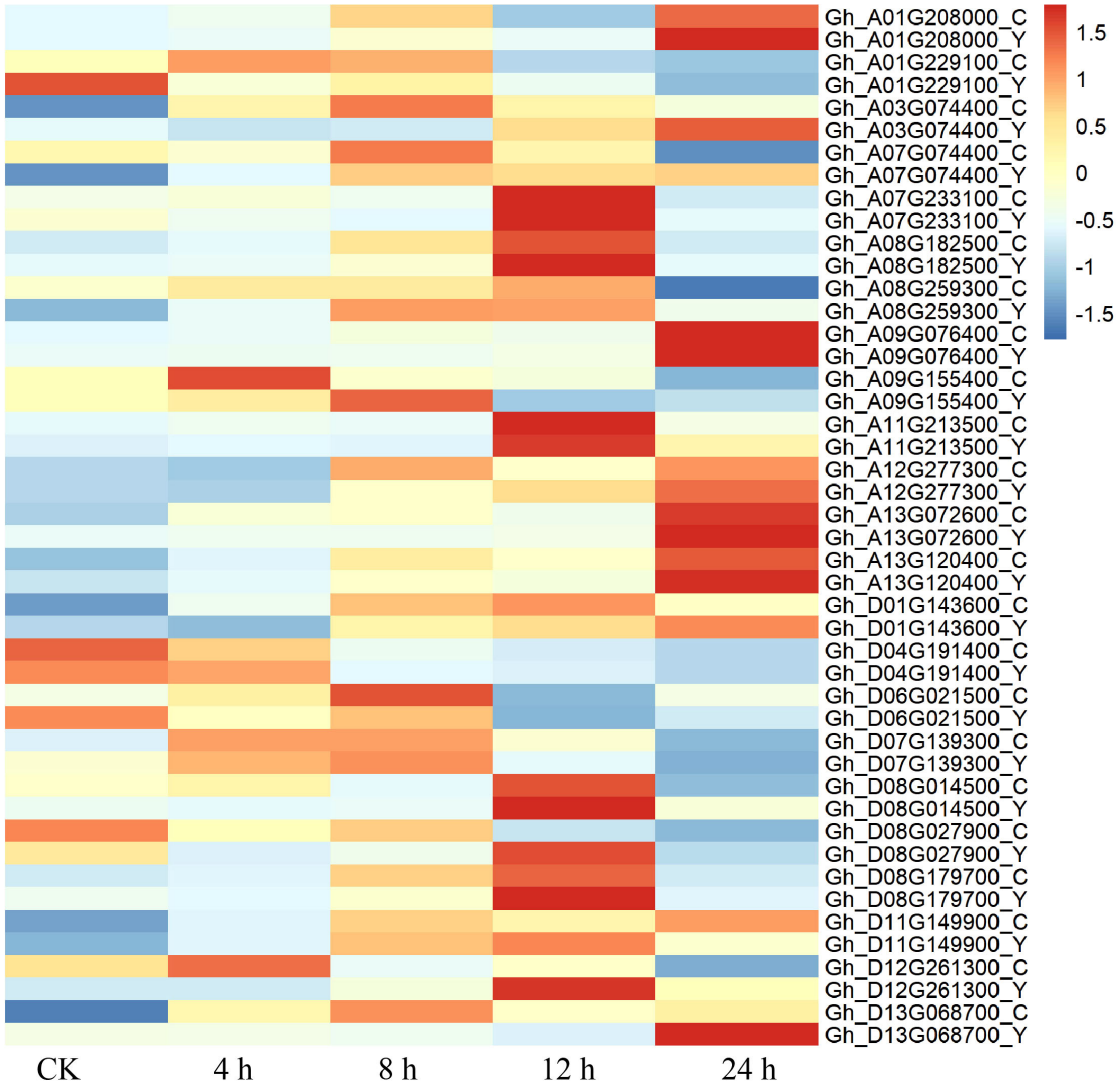
Differential genes in phenylpropanoid biosynthesis pathways.

The analysis of transcript level and metabolite accumulation showed that the major changes of cotton after drought stress involved phenylpropane biosynthesis. The phenylpropanoid biosynthesis pathway is well understood, so genes and metabolites detected by the transcriptome and metabolome are integrated into this metabolic pathway (**Figure 9**). In this pathway, 23 candidate genes were screened. These include CYP84, 4CL1, SNL6, CAD6, PER, and HST, SAMDM, SALAT, CAG, and OMT1 are involved in phenylpropanoid biosynthesis (**Figure 9**). To investigate the underlying mechanisms, we associate DEGs with the above pathways of significant variation. A total of 23 genes were found to be highly related to the phenylpropane biosynthesis pathway (|PCC| > 0.8). We hypothesized that these 23 candidate genes play an important role in phenylpropane-like biosynthesis pathways under drought stress. We concentrated and normalized the genes using FPKM, and found that P450 and methyltransferase genes were more actively expressed in drought-tolerant varieties. In addition, after drought stress, all genes except OMT1, PER43, PER43, SNL6, and SAMDM were up-regulated (**Figure 10**), indicating that they played an important role in drought stress response.

## Discussion

### Transcriptome analysis

A total of 6752, 14460, 22640, 23615, 12311, 15884, 22894 and 25747 DEGs were identified in cotton genotypes R and S under different stress times (**Figure 1**), respectively. The results of GO analysis showed that DEGs was mainly enriched during biosynthesis and hormone metabolism, and the results of this study were consistent with those of wheat under drought stress. KEGG analysis showed that DEGs was mainly enriched in the biosynthesis of secondary metabolites after drought stress. Biosynthesis of cutin, wax and wax; Cysteine and methionine metabolism; Biosynthesis of glucosinolate and so on (**Figure 2**, **3**). Previous studies have shown that the biosynthesis of plant secondary metabolites and the biosynthesis of cutin, wax and wax play an important role in drought stress, and this result is the same in our study (Milena et al., 2020).

### Metabolomics analysis

Metabolomics analysis involves the analysis of endogenous metabolites in organisms, including changes in their type, quantity, and response to external factors (Kell and Oliver, 2016). Compared with other omics analysis methods, metabolomics analysis can better reflect the overall information of organisms under external stimuli. Studies have shown that under drought treatment, plants typically inhibit growth by slowing down metabolic responses, such as carbohydrate interpretation and energy supply, in response to stress (Wei et al., 2013). This conclusion has been fully verified in this study. A total of 1766 DAMS were identified in R and S by metabolomic sequencing, among which lipids and lipid-like molecules, phenylpropanes and polyketones, and organic acids and their derivatives were the three most enriched classes (**Figures 5**, **6**). Lignin biosynthesis and flavonoid bioanabolic pathways play important roles in drought stress of cotton (Yuan et al., 2023). In this study, in addition to flavonoid biosynthesis pathways, lignin biosynthesis and ABC transporters were also significantly enriched, suggesting that these genes may play an important role in drought stress (**Figures 2**, **3**, **8**).

### Phenylpropane metabolic pathway responds to drought stress in cotton

When plants are under drought stress, a large number of metabolic substances will be produced in the body, including the destruction of cell structure, membrane lipid peroxidation, etc., affecting normal growth (Salah et al, 2019). Phenylpropane metabolic pathway plays an important role in cotton response to drought stress. Studies have shown that drought stress can induce the expression of genes related to phenylpropane metabolic pathway in cotton, such as PAL, C4H and 4CL, etc (**Figures 8**, **9**). These genes participate in the synthesis of lignin and flavonoids, and help to enhance the resistance of cotton to drought (Asif et al., 2023; Yuan et al., 2023). Lignin enhances the mechanical strength of the cell wall and forms a physical barrier, while flavonoids have antioxidant and antimicrobial activities. In wheat and maize, phenylpropane metabolic pathways have also been found to improve drought tolerance by regulating osmoregulatory substances and antioxidant enzymes in response to drought stress (Wei et al., 2017; Wang et al., 2022). Moreover, the metabolites of phenylpropane metabolic pathway can also be used as signal molecules to participate in gene expression regulation under drought stress (Shashikumara et al., 2024). In this study, through the combined analysis of transcriptomics and metabolomics, it was found that the accumulation of L-glutamic acid and L-glutathione and other metabolites increased significantly after drought stress. In addition, many DEGs are enriched in the phenylpropane metabolic pathway, and the expression of most genes is up-regulated (**Figures 2**, **3**). These findings suggest that phenylpropane metabolic pathways may play an important role in cotton drought stress response.

## Conclusion

We selected two cotton genotypes (one tolerant to drought stress and one sensitive to drought stress) and placed seedlings under control (normal watering) and drought stress (15% PEG6000) at an early stage of development. Leaf samples were collected and analyzed using a combination of metabolomics and transcriptomics. The expression of different metabolites of cotton under normal and drought stress was analyzed. The enrichment of lipid and phenylpropane biosynthesis was the most significant under drought stress. Cotton responds to drought in many ways, maintaining its metabolism under drought stress by increasing cellular transport enzymes and antioxidant enzymes, and responding to drought stress by regulating phenylpropanoid biosynthesis. The combined analysis of DEG and DAM showed that OMT1, PER43, PER43, SNL6 and SAMDM were closely related to the drought stress response of cotton. The metabolic and transcriptomic findings of this study provide important insights into the growth and development of cotton under drought stress, especially in understanding the mechanisms of cotton response under drought stress. These results provide relevant theoretical basis, and it will help to select excellent cotton varieties that are resistant to drought.

## Data Availability

Transcription sequencing data has been uploaded to the National Genomics Data Center (accession number PRJCA036821).
