# Supplementary material for: Comprehensive transcriptomic and metabolomic analysis revealed drought tolerance regulatory pathways in upland cotton
Source: Front Plant Sci. 2025 Apr 17;16:1571944. doi: 10.3389/fpls.2025.1571944 (PMC12044533; doi:10.3389/fpls.2025.1571944)
Supplement: Supplementary file 1 [file DataSheet1.docx]

Table S1 Transcriptome sequencing data summary statistics

| Sample | Raw Reads | Clean Reads | Clean Base(G) | Error Rate(%) | Q20(%) | Q30(%) | GC Content(%) |
| --- | --- | --- | --- | --- | --- | --- | --- |
| CK_Y_1 | 46691780 | 46099058 | 6.91 | 0.02 | 98.10 | 94.26 | 45.38 |
| CK_Y_2 | 46768654 | 46154778 | 6.92 | 0.03 | 98.07 | 94.18 | 45.34 |
| CK_Y_3 | 50519956 | 49724868 | 7.46 | 0.03 | 98.07 | 94.20 | 45.22 |
| DY_4_1 | 47122578 | 46428720 | 6.96 | 0.03 | 97.92 | 93.86 | 45.26 |
| DY_4_2 | 49796906 | 49102642 | 7.37 | 0.03 | 98.00 | 94.03 | 44.96 |
| DY_4_3 | 47381848 | 46740098 | 7.01 | 0.03 | 97.66 | 93.29 | 44.84 |
| DY_8_1 | 48485096 | 47793102 | 7.17 | 0.03 | 98.06 | 94.15 | 44.24 |
| DY_8_2 | 47162382 | 46518318 | 6.98 | 0.03 | 98.02 | 94.04 | 43.98 |
| DY_8_3 | 47873120 | 47220616 | 7.08 | 0.03 | 97.94 | 93.87 | 44.09 |
| DY_12_1 | 45202472 | 44501734 | 6.68 | 0.03 | 97.94 | 93.88 | 44.50 |
| DY_12_2 | 47279110 | 46639300 | 7.00 | 0.02 | 98.09 | 94.25 | 44.30 |
| DY_12_3 | 45720788 | 44965886 | 6.74 | 0.03 | 98.02 | 94.07 | 44.30 |
| DY_24_1 | 46733492 | 46101438 | 6.92 | 0.03 | 98.04 | 94.11 | 44.64 |
| DY_24_2 | 52809496 | 52043036 | 7.81 | 0.03 | 98.04 | 94.11 | 44.32 |
| DY_24_3 | 50126800 | 49351054 | 7.40 | 0.03 | 98.06 | 94.14 | 44.21 |
| CK_C_1 | 52525346 | 51814864 | 7.77 | 0.02 | 98.10 | 94.24 | 44.88 |
| CK_C_2 | 52184538 | 51497038 | 7.72 | 0.02 | 98.13 | 94.31 | 45.04 |
| CK_C_3 | 54519360 | 53788070 | 8.07 | 0.03 | 98.09 | 94.21 | 44.91 |
| DC_4_1 | 48667728 | 47935452 | 7.19 | 0.03 | 97.93 | 93.92 | 45.13 |
| DC_4_2 | 43982818 | 43349942 | 6.50 | 0.03 | 97.95 | 93.93 | 44.78 |
| DC_4_3 | 43471586 | 42909014 | 6.44 | 0.02 | 98.19 | 94.50 | 44.71 |
| DC_8_1 | 43848752 | 43198340 | 6.48 | 0.02 | 98.20 | 94.57 | 44.85 |
| DC_8_2 | 47489908 | 46780164 | 7.02 | 0.02 | 98.25 | 94.64 | 44.84 |
| DC_8_3 | 46347378 | 45646948 | 6.85 | 0.02 | 98.25 | 94.67 | 44.51 |
| DC_12_1 | 45544110 | 44872948 | 6.73 | 0.02 | 98.20 | 94.56 | 45.00 |
| DC_12_2 | 48899074 | 48320236 | 7.25 | 0.03 | 97.94 | 93.88 | 44.67 |
| DC_12_3 | 43483658 | 42775522 | 6.42 | 0.03 | 98.04 | 94.15 | 44.28 |
| DC_24_1 | 41734540 | 41126702 | 6.17 | 0.03 | 97.98 | 93.97 | 44.40 |
| DC_24_2 | 44764148 | 44079356 | 6.61 | 0.03 | 97.74 | 93.49 | 44.21 |
| DC_24_3 | 48675544 | 47970936 | 7.20 | 0.03 | 97.61 | 93.20 | 44.47 |


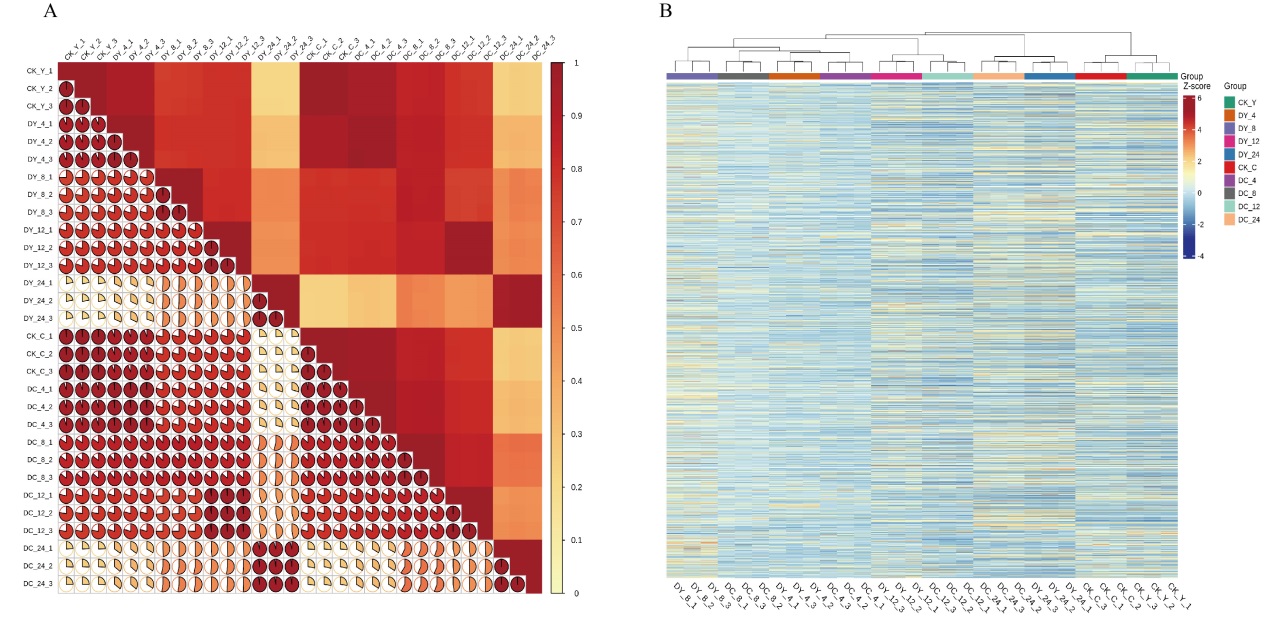


Figure S1. Quantitative analysis of gene expression in cotton under drought stress. A: Correlation heat map,B: differential gene clustering heat map.


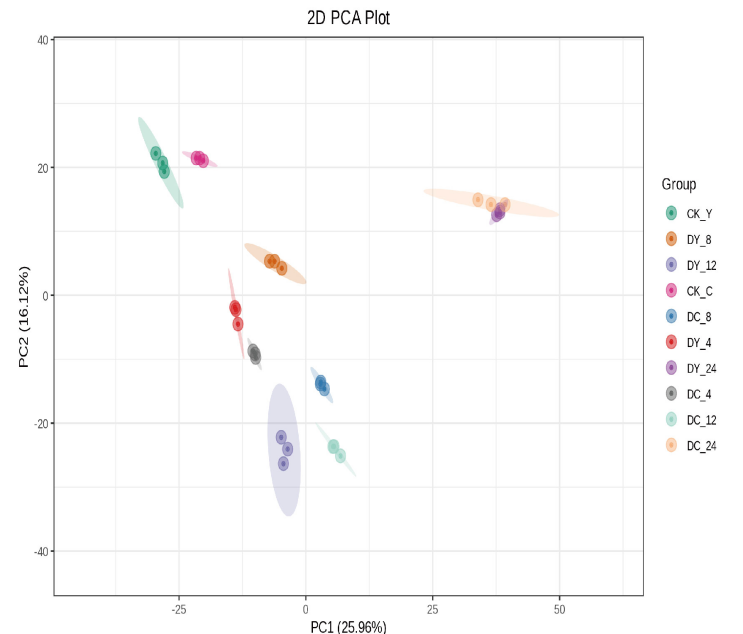


Figure S2. PCA analysis of cotton metabolome under drought stress.
